# Effects of meteorological factors on the incidence of mumps and models for prediction, China

**DOI:** 10.1186/s12879-020-05180-7

**Published:** 2020-07-02

**Authors:** Wen-ting Zha, Wei-tong LI, Nan Zhou, Jia-jia Zhu, Ruihua Feng, Tong Li, Yan-bing Du, Ying Liu, Xiu-qin Hong, Yuan Lv

**Affiliations:** Key Laboratory of Molecular Epidemiology of Hunan Province, School of Medicine, Hunan Normal University, Changsha, Hunan People’s Republic of China 410081

**Keywords:** Mumps, Meteorological factors, ARIMA, ARIMAX, Prediction effect

## Abstract

**Background:**

Mumps is an acute respiratory infectious disease with obvious regional and seasonal differences. Exploring the impact of climate factors on the incidence of mumps and predicting its incidence trend on this basis could effectively control the outbreak and epidemic of mumps.

**Methods:**

Considering the great differences of climate in the vast territory of China, this study divided the Chinese mainland into seven regions according to the administrative planning criteria, data of Mumps were collected from the China Disease Prevention and Control Information System, ARIMA model and ARIMAX model with meteorological factors were established to predict the incidence of mumps.

**Results:**

In this study, we found that precipitation, air pressure, temperature, and wind speed had an impact on the incidence of mumps in most regions of China and the incidence of mumps in the north and southwest China was more susceptible to climate factors. Considering meteorological factors, the average relative error of ARIMAX model was 10.87%, which was lower than ARIMA model (15.57%).

**Conclusions:**

Meteorology factors were the important factors which can affect the incidence of mumps, ARIMAX model with meteorological factors could better simulate and predict the incidence of mumps in China, which has certain reference value for the prevention and control of mumps.

## Background

Mumps, commonly caused by mumps virus (MuV), is an acute respiratory infectious disease characterized by the swelling of parotid gland, localized pain, and fever [1]. Mumps are mainly transmitted by droplets and direct contact, which are infectious 7 days before and 9 days after parotid gland enlargement [2]. A number of countries around the world have reported cases of mumps and China was one of the areas with high incidence of mumps [4]. From the year 2004 to 2013, the total incidence of mumps in China reached 24.2/100,000, among which the reported incidence of children aged 5–9 was 118.2–281.4/100,000 [4]. In recent years, the incidence of mumps has been decreasing, however in some certain conditions people are still at high risk of being infected. For example, children in gathering places, as well as someone living in crowded and poor sanitation [5].

The incidences of mumps have an obvious differences from the regional and seasonal dimension. In the tropical regions of the world, mumps occurs all year round, while it is just common during spring and winter in the temperate regions [3]. Nowadays, the global climate has been facing dramatic changes and the extreme weather is more frequent. Thus exploring the impact of climate factors on the incidence of mumps and predicting its incidence are contributive to control the outbreak and epidemic of mumps [6]. Autoregressive integrated moving average (ARIMA) is often used to explore the regular pattern of disease development from the time dimension in the field of medicine. Besides, ARIMAX model of multivariate time series adds other variables related to the research sequence as input variables in order to make more accurate prediction [7–10]. In this study, ARIMA and ARIMAX model with meteorological factors were established, and the relationship between mumps and meteorology factors in different regions of China have been explored, the comparison of the two models has provide reference for both prevention and control of mumps and the effective prediction of other infectious diseases in China.

## Methods

### The sources of data

The epidemic surveillance data of mumps from January 2006 to December 2016 in provinces, municipalities directly under the Central Government, and autonomous regions of China were collected from the Public Health Science Data Center of China Disease Prevention and Control Information System (http://www.phsciencedata.cn/Share). The surveillance coverage of mumps was unchanged from 2006 to 2016, and all case reports were based on the confirmed diagnosis of mumps [4]. In China, all medical institutions, Centers for Disease Control and Prevention (CDC), blood collection and supply institutions were responsible reporting units of infectious diseases. Doctors in school, community or hospital who have diagnosed mumps for the first time should submit a report card, at the same time, the staff in infectious disease management department should directly report the case on the network, or send the report card to local CDC within the prescribed time. Mumps is legally classified as C level infectious disease in China, which means the report should be submitted within 24 h once the diagnosis is confirmed.

The demographic data of different regions were from the National Bureau of Statistics; National meteorological monitoring data, including average precipitation (mm), average air pressure (hPa), average temperature (°C), average relative humidity (%), minimum and maximum temperature (°C), days with daily precipitation ≥0.1 mm and maximum wind speed (ms)), were from the National Meteorological Information Center (http://data.cma.cn).

### Data preprocessing

Considering the great differences of climate in the vast territory of China, this study has divided the Chinese mainland into seven regions according to the administrative planning criteria, i.e. North China (includes Beijing, Tianjin, Hebei, Shanxi, Inner Mongolia), Northeast China (includes Liaoning, Jilin, Heilongjiang), East China (includes Shanghai, Jiangsu, Zhejiang, Anhui, Fujian, Jiangxi, Shandong), Central China (includes Henan, Hubei, Hunan), Southwest China (includes Chongqing, Sichuan, Guizhou) Prefecture, Yunnan, Tibet, Northwest China (includes Shaanxi, Gansu, Qinghai, Ningxia, Xinjiang), South China (includes Guangdong, Guangxi, Hainan). (Supplement 1).

The incidence of mumps was calculated by dividing the number of reported cases per month by the average population of the same period in the region, and the meteorological factors in different regions were described by the average monthly data of all observation points in the region.

### ARIMA model

Data of mumps and meteorological factors in different regions were divided into two parts from the perspective of time. One part was from January 2006 to December 2015, which was used to fit the model of mumps incidence; another part was from January to December 2016, which was aimed to evaluate the prediction effect of the optimal model.

The model could be expressed as ARIMA (p, d, q) × (P, D, Q) s, where d and D showed the order of ordinary difference and seasonal difference, which were the data conversion methods used to transform original time series into stable time series; p and q showed the order of autoregression and moving average in continuous model respectively; P and Q showed the order of autoregression and moving average in seasonal model respectively; the subscripted letter “s” showed the seasonal period length, in this research, s = 12.

The process of building ARIMA model of mumps was as follows [11]:

1. Model stationarization: Drew time series graph of mumps incidence data. When the graph was non-stationary, it needed to be stabilized by data conversion like means of difference, seasonal difference, logarithmic and exponential transformation of original data.
2. Model recognition: Judged the order of model and estimated the range of p, d, P, D according to the graph characteristics by drawing the autocorrelation function (ACF) and partial autocorrelation function (PACF) of mumps.
3. Parameter estimation: The least square method was used to estimate the parameters in the autoregressive process and moving average process, the significance of the estimated model parameters were tested at the same time, where α = 0.05.
4. Model Testing: Firstly, calculated the residual between the real value and the model fitting value, then formed the residual sequence. Secondly, used the Ljung-Box Q test (LBQ test) to determine whether the model was a white noise sequence or not, which means the model was sufficient to extract data information. Thirdly, determined the optimal model according to Schwarz Bayesian criterion (SBC), the smaller the SBC value was, the better the fitting effect of the model was.
5. Model prediction: Predicted the incidence of mumps in different regions from January to December 2016, then judged the prediction effect of this model by comparing with the actual reported incidence.

### ARIMAX model

ARIMAX model is an extension of ARIMA modelling incorporating an explanatory independent variable. An ARMAX model could simply be regard as a multiple regression with one or more AR and MA terms [12]. The corresponding residual white noise sequence was obtained by establishing a one-element time series model for each individual meteorological variable. Based on the cross-correlation function (CCF) of residual white noise, the meteorological factors which affects the incidence of mumps were found out, and the optimal lag time was obtained. The selected meteorological factors were incorporated into the previously determined time series model to construct multivariate time series ARIMAX model. The optimal ARIMAX model was determined according to the minimum criterion of SBC. Lastly, compared the prediction effect of ARIMA and ARIMAX model with the relative error between actual and predicted incidence.

### Statistic software

Using Microsoft Excel 2010 to establish the original database, using IBM SPSS statistics 23.0 for statistical analysis.

## Results

### Epidemiological characteristics of mumps in China from 2006 to 2016

From 2006 to 2016, 3.2 million cases of mumps were reported in China, which declined year by year after the highest average monthly incidence rate in 2012. The peak seasons of mumps in China were spring and winter, which accounted for 30.00 and 30.06% in 1 year respectively. Mumps occured in all provinces of China every year, mostly were concentrated except for north of China, which was shown in Table 1.

**Table 1:** Epidemiological characteristics of mumps in China from 2006 to 2016

Variable	Number of cases	Average monthly incidence (1/100,000) or constituent ratio (%)
**Year**^a^		
2006	271,397	20.65
2007	252,701	19.13
2008	310,826	23.41
2009	299,329	22.43
2010	298,932	22.29
2011	454,385	33.72
2012	479,518	35.59
2013	327,759	24.09
2014	187,500	13.71
2015	182,833	13.30
2016	175,001	12.66
**Season**^b^		
Spring	974,806	30.08
Summer	707,817	21.84
Autumn	585,364	18.07
Winter	972,194	30.00
**Regions**^b^		
North China	317,528	9.80
East China	792,047	24.44
South China	495,888	15.30
Central China	497,570	15.36
Southwest	556,687	17.18
Northwest	382,676	11.81
Northeast	197,785	6.10

Notes: ^a^Average monthly incidence (1/100,000), ^b^constituent ratio (%)

### ARIMA model

**Fig. 1:**
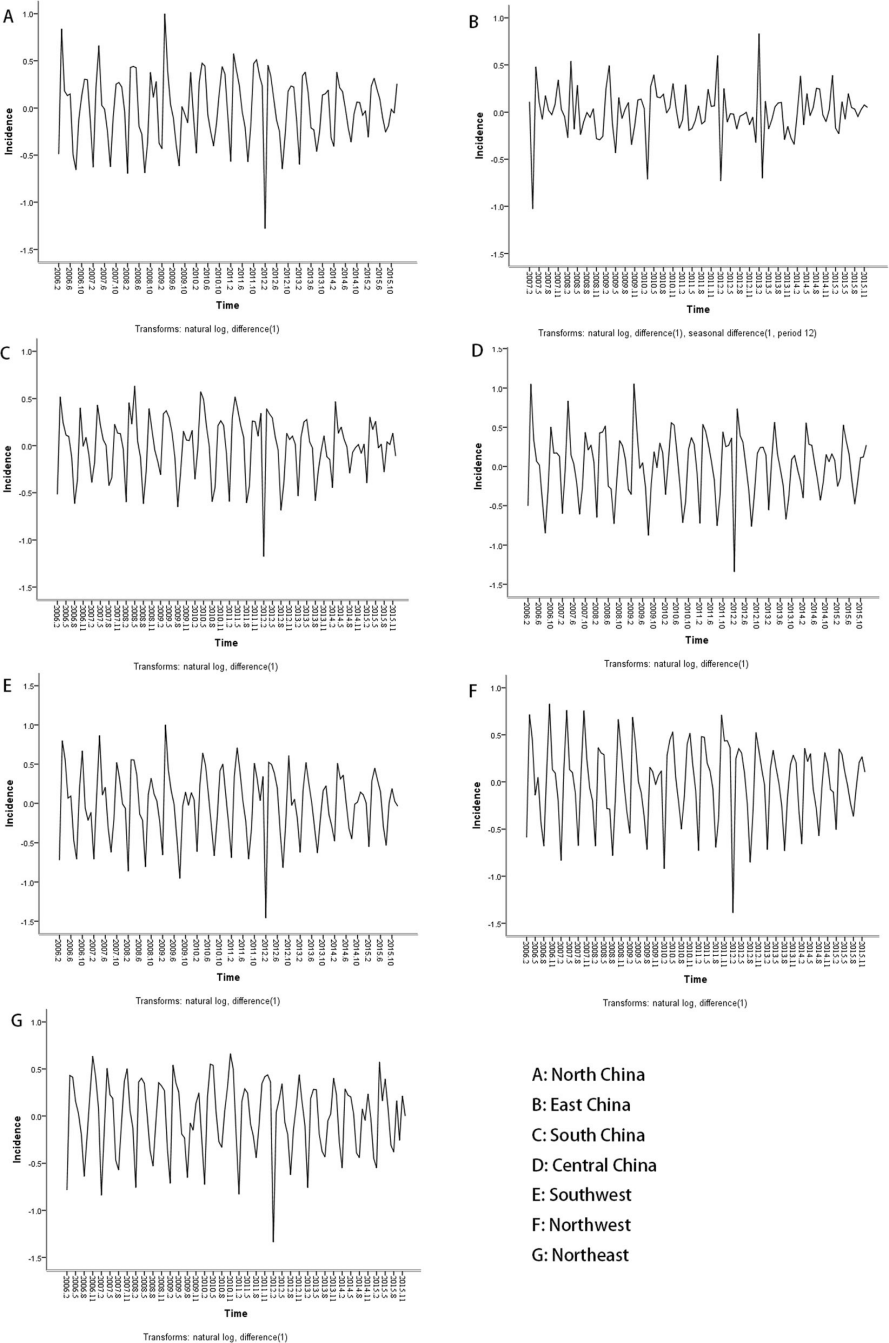
The stationary time series of mumps incidence in different regions after conversion

**Fig. 2:**
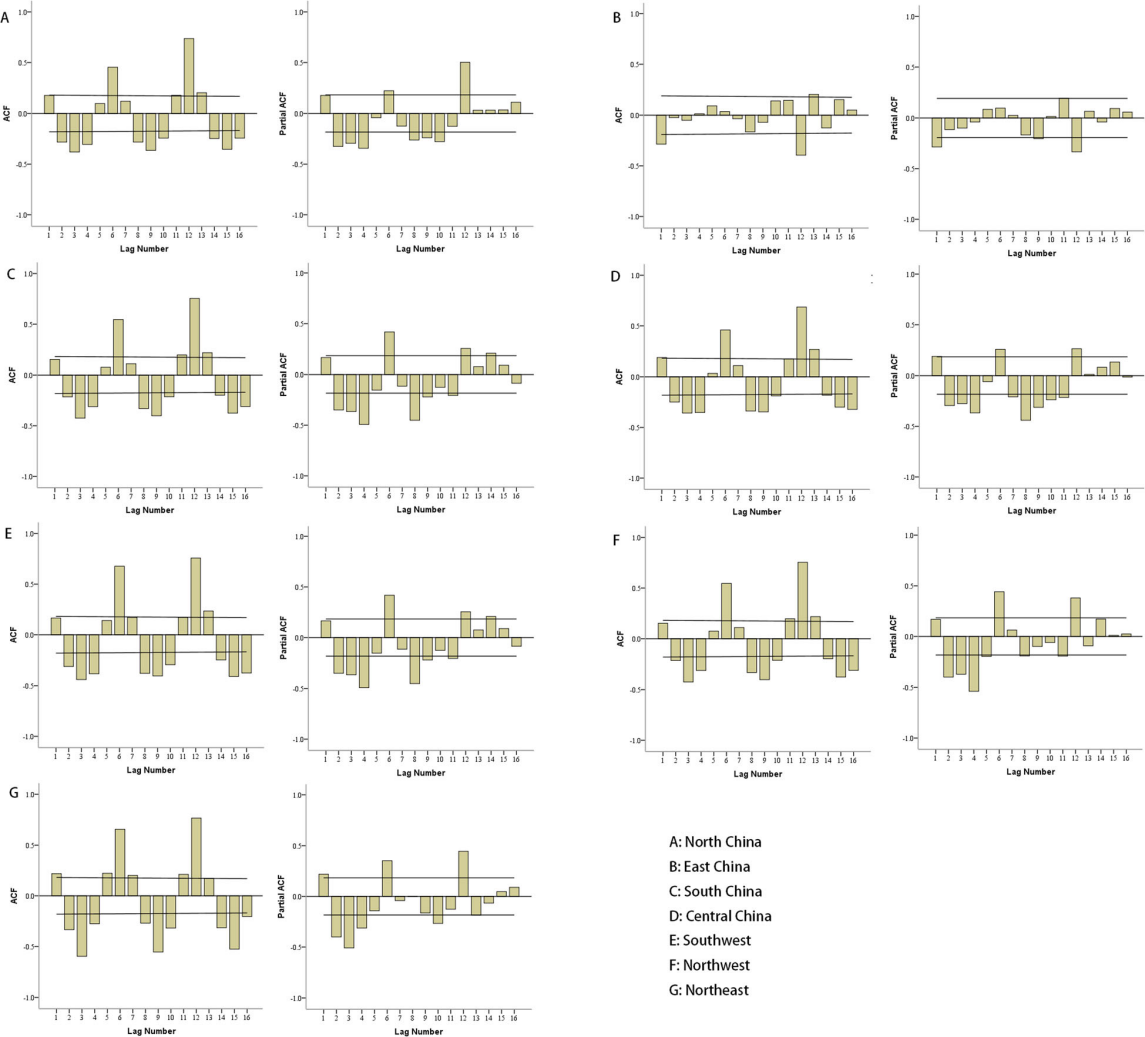
The ACF and PACF diagrams of models in different regions

The original sequence of mumps incidence in different regions were non-stationary time series (Supplement 2). The original sequence of mumps incidence in different regions were converted into stationary time series by logarithmic transformation, ordinary difference or seasonal difference (Fig. 1). The parameter of models in different regions were firstly estimated according to the characteristics of time series diagram, ACF, and PACF diagrams (Figs. 1 and 2). The optimal ARIMA model in different regions was the one with the smallest SBC by fitting p, q, P, Q of 0, 1 and 2 in order (Table 2). LBQ test showed that models in different regions conformed to the white noise sequence conditions (*P* > 0.05), which means that the optimal models could extract information sufficiently (Table 2).

**Table 2:** The optimal ARIMA model in different regions of China

Regions	Model	Fitting effect			LBQ test	
		R^2^	Stable R^2^	SBC	Q	P
North China	(0,1,0) (1,0,1)_12_	0.820	0.830	−1.663	14.257	0.506
East China	(0,1,0) (0,1,1)_12_	0.858	0.389	− 1.836	22.050	0.107
South China	(0,1,0) (1,0,0)_12_	0.884	0.108	−1.127	25.607	0.082
Central China	(0,1,0) (1,0,1)_12_	0.856	0.393	−1.767	17.171	0.375
Southwest	(0,1,0) (2,0,1)_12_	0.854	0.700	−1.327	22.808	0.088
Northwest	(1,1,1) (0,0,0)_12_	0.814	0.818	−0.109	22.237	0.102
Northeast	(0,1,0) (1,0,1)_12_	0.822	0.166	−1.749	14.557	0.627

Predicted the average monthly incidence of mumps from January to December 2016 with ARIMA model, we found that the predicted values were basically in agreement with the actual incidence values. All the actual values were within the 95% confidence interval of the predicted values and the average relative error was 15.57% (Table 3).

**Table 3:** The prediction effect of ARIMA and ARIMAX model in different regions

Region	Month	Actual incidence (1/100,000)	ARIMA		ARIMAX	
			Predicting incidence (95%CI)	Relative error (%)	Predicting incidence (95%CI)	Relative error (%)
North China	Jan	0.79	0.83(0.57–1.17)	5.06	0.82(0.57 ~ 1.20)	3.80
	Feb	0.52	0.51(0.31–0.81)	1.92	0.52(0.33 ~ 0.69)	0.00
	Mar	0.72	0.82(0.44–1.39)	13.89	0.79(0.34 ~ 1.32)	9.72
	Apr	0.94	1.21(0.61–2.17)	28.72	1.18(0.43 ~ 2.07)	25.53
	May	1.22	1.53(0.73–2.83)	25.41	1.40(0.67 ~ 2.62)	14.75
	Jun	1.30	1.52(0.70–2.90)	16.92	1.48(0.68 ~ 2.85)	13.85
	Jul	1.11	1.25(0.56–2.44)	12.61	1.20(0.43 ~ 2.38)	8.11
	Aug	0.90	0.81(0.35–1.62)	10.00	0.80(0.44 ~ 1.78)	11.11
	Sep	0.85	0.71(0.30–1.43)	16.47	0.73(0.45 ~ 1.67)	14.12
	Oct	0.79	0.84(0.35–1.70)	6.33	0.81(0.23 ~ 1.63)	2.53
	Nov	0.99	1.00(0.41–2.04)	11.11	1.00(0.40 ~ 2.27)	1.01
	Dec	1.21	1.26(0.51–2.60)	4.13	1.19(0.51 ~ 2.52)	1.65
East China	Jan	0.83	0.89(0.59–1.28)	7.23	0.85(0.61 ~ 1.25)	2.41
	Feb	0.54	0.53(0.30–0.88)	1.85	0.48(0.32 ~ 0.74)	11.11
	Mar	0.81	0.87(0.42–1.60)	7.41	0.74(0.35 ~ 1.66)	8.64
	Apr	1.04	1.35(0.58–2.69)	29.81	1.07(0.61 ~ 2.01)	2.88
	May	1.34	1.81(0.70–3.89)	35.07	1.45(0.57 ~ 2.58)	8.21
	Jun	1.39	1.85(0.65–4.23)	33.09	1.49(0.62 ~ 2.98)	7.19
	Jul	1.25	1.47(0.47–3.55)	17.60	1.10(0.43 ~ 2.57)	12.00
	Aug	0.93	0.80(0.23–2.04)	13.98	1.02(0.24 ~ 1.47)	9.68
	Sep	0.90	0.64(0.17–1.69)	28.89	0.85(0.21 ~ 1.45)	5.56
	Oct	0.84	0.67(0.17–1.87)	20.24	0.70(0.18 ~ 1.59)	16.67
	Nov	0.82	0.75(0.17–2.18)	8.54	0.79(0.19 ~ 1.54)	3.66
	Dec	0.79	0.94(0.20–2.83)	18.99	0.83(0.22 ~ 1.98)	5.06
South China	Jan	1.33	1.36(0.91–1.94)	2.26	–	–
	Feb	0.78	0.79(0.57–1.40)	1.28	–	–
	Mar	1.15	1.26(0.73–2.02)	9.57	–	–
	Apr	1.40	1.50(0.82–2.53)	7.14	–	–
	May	1.66	1.96(1.01–3.44)	18.07	–	–
	Jun	2.17	1.93(0.95–3.52)	11.06	–	–
	Jul	2.27	1.97(0.92–3.74)	13.22	–	–
	Aug	1.61	1.50(0.67–2.94)	6.83	–	–
	Sep	1.60	1.57(0.67–3.17)	1.88	–	–
	Oct	1.79	1.60(0.65–3.33)	10.61	–	–
	Nov	1.87	1.84(0.72–3.94)	1.60	–	–
	Dec	2.01	1.66(0.62–3.65)	17.41	–	–
Central China	Jan	1.83	1.86(1.21–2.75)	1.64	1.85(1.36 ~ 1.52)	1.09
	Feb	1.03	1.07(0.64–1.70)	3.88	1.06(0.47 ~ 1.68)	2.91
	Mar	1.15	1.19(0.88–2.67)	3.48	1.30(0.79 ~ 2.10)	13.04
	Apr	1.58	2.01(1.33–4.56)	27.33	1.89(0.86 ~ 2.40)	19.62
	May	2.20	2.58(1.65–6.28)	17.27	2.40(1.92 ~ 5.67)	9.09
	Jun	2.46	3.56(1.62–6.85)	44.72	3.35(1.92 ~ 6.04)	36.18
	Jul	2.37	2.83(1.22–5.66)	19.41	2.21(1.14 ~ 4.07)	6.75
	Aug	1.48	1.48(0.61–3.06)	0.00	1.31(0.71 ~ 2.45)	11.49
	Sep	1.26	1.12(0.44–2.40)	11.11	1.19(0.56 ~ 2.28)	5.56
	Oct	1.62	1.43(0.53–3.16)	11.73	1.51(0.68 ~ 2.93)	6.79
	Nov	2.22	1.71(0.60–3.89)	22.97	1.56(0.62 ~ 3.21)	29.73
	Dec	3.30	2.11(0.71–4.92)	36.06	2.34(0.63 ~ 3.45)	29.09
South west	Jan	1.17	1.26(0.77–1.99)	7.69	1.20(0.82 ~ 1.69)	2.56
	Feb	0.59	0.70(0.40–1.14)	18.64	0.63(0.53 ~ 1.38)	6.78
	Mar	1.06	0.96(0.51–1.67)	9.43	1.04(0.54 ~ 1.71)	1.89
	Apr	1.41	1.60(0.79–2.89)	13.48	1.46(0.73 ~ 1.55)	3.55
	May	1.89	2.20(1.05–4.09)	16.40	2.21(1.06 ~ 4.10)	16.93
	Jun	1.95	2.43(1.13–4.62)	24.62	2.51(1.23 ~ 4.61)	28.72
	Jul	1.69	1.84(0.84–3.55)	8.88	1.85(0.89 ~ 3.45)	9.47
	Aug	1.18	1.07(0.48–2.08)	9.32	1.12(0.61 ~ 2.14)	5.08
	Sep	1.28	1.00(0.44–1.96)	21.88	1.01(0.47 ~ 1.93)	21.09
	Oct	1.55	1.18(0.52–2.33)	23.87	1.20(0.57 ~ 2.29)	22.58
	Nov	1.57	1.36(0.59–2.68)	13.38	1.39(0.54 ~ 2.68)	11.46
	Dec	1.58	1.29(0.56–2.56)	18.35	1.31(0.59 ~ 2.55)	17.09
North west	Jan	1.59	1.55(1.00–2.29)	2.52	1.64(1.13 ~ 2.46)	3.14
	Feb	0.89	0.90(0.49–1.52)	1.12	0.89(0.51 ~ 1.49)	0.00
	Mar	1.17	1.26(0.62–2.39)	7.69	1.34(0.64 ~ 2.38)	14.53
	Apr	1.45	1.76(0.77–3.45)	21.38	1.63(0.76 ~ 3.19)	12.41
	May	1.84	2.16(0.89–4.46)	17.39	2.06(0.89 ~ 4.01)	11.96
	Jun	1.93	2.13(0.82–4.56)	10.36	1.90(0.76 ~ 3.98)	1.55
	Jul	1.53	1.71(0.63–3.78)	11.76	1.64(0.63 ~ 3.45)	7.19
	Aug	1.28	1.06(0.38–2.40)	17.19	1.04(0.36 ~ 2.28)	18.75
	Sep	1.31	1.01(0.35–2.33)	22.90	1.10(0.36 ~ 2.22)	16.03
	Oct	1.41	1.30(0.43–3.05)	7.80	1.26(0.44 ~ 2.89)	10.64
	Nov	2.26	1.70(0.55–4.05)	24.78	1.83(0.61 ~ 3.94)	19.03
	Dec	2.29	1.85(0.59–4.47)	19.21	1.96(0.71 ~ 4.50)	14.41
North east	Jan	0.44	0.37(0.25–0.52)	15.91	0.39(0.29 ~ 0.54)	11.36
	Feb	0.29	0.18(0.10–0.30)	37.93	0.20(0.13 ~ 0.30)	31.03
	Mar	0.49	0.26(0.13–0.46)	46.94	0.32(0.26 ~ 0.55)	34.69
	Apr	0.49	0.34(0.15–0.65)	30.61	0.39(0.20 ~ 0.69)	20.41
	May	0.72	0.47(0.19–0.97)	34.72	0.62(0.29 ~ 1.18)	13.89
	Jun	0.70	0.47(0.17–1.04)	32.86	0.66(0.29 ~ 1.32)	5.71
	Jul	0.53	0.35(0.12–0.81)	33.96	0.52(0.23 ~ 1.10)	1.89
	Aug	0.43	0.22(0.07–0.55)	48.84	0.29(0.14 ~ 0.66)	32.56
	Sep	0.46	0.23(0.07–0.61)	50.00	0.30(0.12 ~ 0.71)	34.78
	Oct	0.40	0.24(0.06–0.64)	40.00	0.26(0.10 ~ 0.59)	35.00
	Nov	0.44	0.33(0.08–0.93)	25.00	0.33(0.10 ~ 0.85)	25.00
	Dec	0.46	0.39(0.09–1.12)	15.22	0.43(0.13 ~ 1.09)	6.52

Note: There were no meteorological factors related to the incidence of mumps in south China

### ARIMAX model

**Fig. 3:**
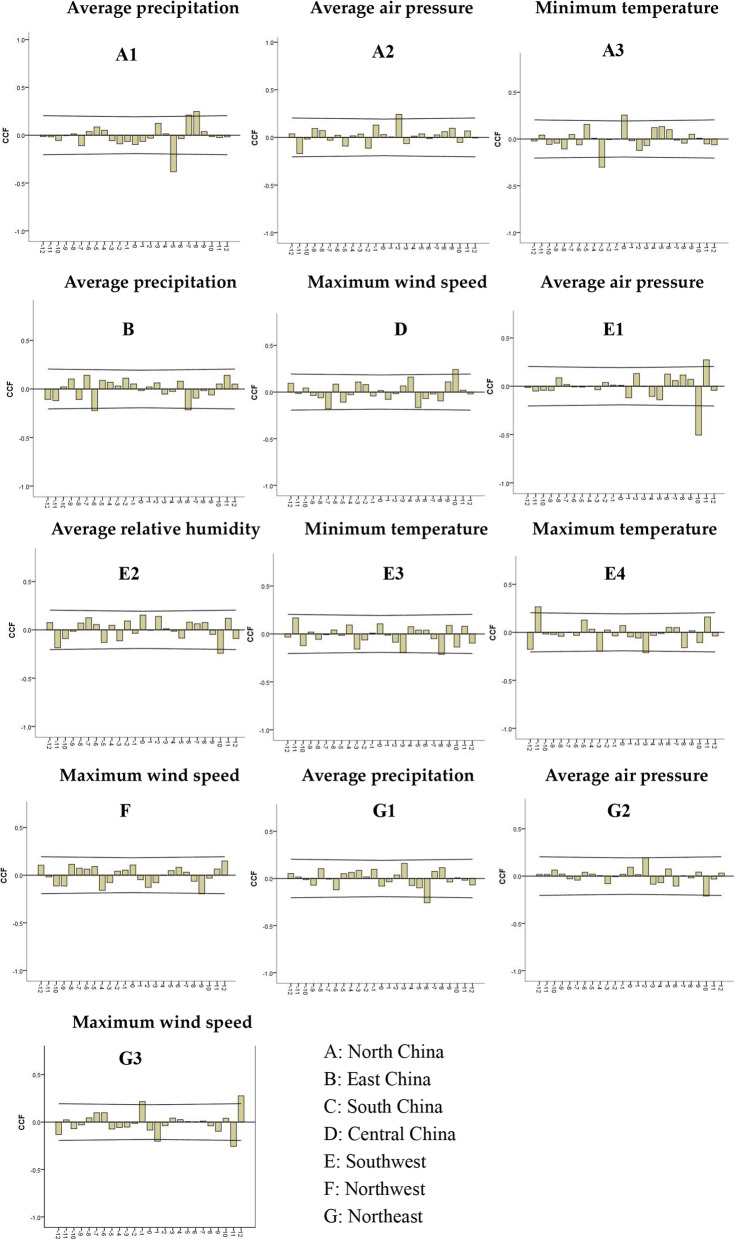
Cross correlation analysis between meteorological factors and mumps incidence in different regions

The optimal ARIMA model of each meteorological factor sequence was used to filter the differential meteorological factor sequence and mumps sequence after the difference, and then calculated the co-correlation coefficient (CCF) between the meteorological factors and the mumps incidence sequence (Fig. 3). The CCF chart lagged a certain order beyond the confidence interval, indicating that the incidence of mumps is related to this meteorological factor. Considering the lag of the 0–12 order, we found that the incidence of mumps was correlated with average precipitation (lags 5 or 8), average air pressure (lags 2) and minimum temperature (lags 0) in north China; correlated with average precipitation (lags 6) in east China; correlated with maximum wind speed (lags 10) in central of China; correlated with average air pressure (lags 10 or 11), average relative humidity (lags 10), minimum temperature (lags 8) and maximum temperature (lags 3) in Southwest; correlated with maximum wind speed (lags 9) in the northwest; correlated with average precipitation (lags 6), average air pressure (lags 10) and maximum wind speed (lags 1 or 11 or 12) in the northeast. No meteorological factors related to the incidence of mumps in south China (Fig. 3, Table 4).

**Table 4:** Relationship between mumps and meteorological factors in different regions

Regions	Meteorological factors	Lag	Correlation
North China	Average precipitation	5	P
		8	N
	Average air pressure	2	P
	Minimum temperature	0	P
East China	Average precipitation	6	N
Central China	Maximum wind speed	10	P
Southwest	Average air pressure	10	N
		11	P
	Average relative humidity	10	N
	Minimum temperature	8	N
	Maximum temperature	3	N
Northwest	Maximum wind speed	9	N
Northeast	Average precipitation	6	N
	Average air pressure	10	N
	Maximum wind speed	1	N
		11	N
		12	P
South China	–	–	–

*P* Positive correlation, *N* Negative correlation

Established the ARIMAX model by incorporating significant statistical relevant meteorological factors into the ARIMA model, the LBQ test showed that ARIMAX models in different regions conformed to the white noise sequence conditions (*P* > 0.05) (Supplement 3). Predicted the incidence of mumps from January to December 2016 with ARIMAX model, we found that the predicted values were basically consistent with the actual incidence values. All the actual values were within the 95% confidence interval of the predicted values, with an average relative error of 10.87%, which was lower than ARIMA model (Table 3). Supplement 3.

## Discussion

With the increase of global temperature and extreme weather events, it is important to research and predict the impact of meteorological factors on the incidence of diseases. Mumps is an acute infectious disease, which has a great impact on the physical and mental health of adolescents in China. In this study, we found that precipitation, air pressure, temperature, and wind speed had an effect on the incidence of mumps in most regions of China, which was consistent with the studies in Japan and Taiwan [12, 13]. Temperature and precipitation may affect the survival environment and transmission routes of pathogens, as well as exposure opportunities and sensitivity of susceptible populations [12, 14, 15]. Warm and humid weather are conducive to virus reproduction and evolution, and in warm weather, children are prone to go outdoors, which may increase the possibility of infection [12, 15]. In most regions of China, air pressure was negatively correlated with the incidence of mumps, a possible explanation could be that, the low air pressure causes the thin air condition, which was the reason for a low partial blood pressure of oxygen in human beings and then results in the reduction of resistance of the human body [16]. The acceleration of wind speed may speed up the flow of virus aerosol and expand the coverage of mumps, at low pressure and high wind speed, the virus was easy to spread and cause infection [12, 15, 16].

Mumps occured in all provinces of China every year, most of which were concentrated except for north of China, but the incidence of mumps in north and southwest China were more susceptible to climate factors, which was related to the meteorological characteristics in various regions. In the east, south and central of China, the rainwater was abundant, the weather was warm and humid; in the west of China, the air pressure was low and the air was thin, so these areas were more susceptible to mumps [17]. In the north and southwest China, the temperature and wind speed usually changed rapidly, and there was more extreme weather, which was more susceptible to the influence of meteorological factors.

Predicted the incidence of mumps from January to December 2016 with the ARIMA model, we found that the predicted values were basically in agreement with the actual incidence values, with an average relative error of 15.57%. Established ARIMAX model through incorporating statistical significant relevanted meteorological factors into the ARIMA model, we found that the predicted values were also basically in agreement with the actual incidence values, with an average relative error of 10.87%, which was lower than ARIMA model. Considering meteorological factors, ARIMAX model could better simulate and predict the incidence of mumps in China, which has certain reference value for the prevention and control of mumps.

Although two models in this study (ARIMA and ARIMAX) showed a good predictive effect on the incidence of mumps in China, they have not taken the economic and demographic factors into account, so in the following study, we would try to integrate more factors which may affect the incidence of mumps into models for comprehensive analysis. Besides, the ARIMA and ARIMAX model applied the historical epidemic data of mumps, with the occurrence of new cases, we should constantly adjust the parameters of the models to improve the sensitivity and accuracy of prediction, so as to make the research results closer to the actual work of prevention and control [14, 18].

## Conclusions

Precipitation, air pressure, temperature, wind speed had an impact on the incidence of mumps in most regions of China and the incidence of mumps in north and southwest China, were more susceptible to climate factors. Considering meteorological factors, the average relative error of ARIMAX model was lower than ARIMA model; ARIMAX model could better simulate and predict the incidence of mumps in China, which has certain reference value for the prevention and control of mumps.

## Supplementary information

**Additional file 1: Supplement 1.** Schematic diagram of the division of different regiousnin mainland China.

**Additional file 2: Supplement 2.** Time sequence diagram of mumps incidence in different regions.

**Additional file 3: Supplement 3.** The optimal ARIMAX model for each meteorological sequence in different regions of China.

## Data Availability

All data and material in our study were availability. The epidemic surveillance data of mumps were from the Public Health Science Data Center of China Disease Prevention and Control Information System (http://www.phsciencedata.cn/Share), the demographic data of different regions were from the National Bureau of Statistics (http://www.stats.gov.cn), National meteorological monitoring data were from the National Meteorological Information Center (http://data.cma.cn), all data are open to the public.

## Abbreviations

MuV: Mumps virus
ARIMA: Autoregressive integrated moving average
ACF: Autocorrelation function
PACF: Partial autocorrelation function
LBQ test: Ljung-Box Q test
SBC: Schwarz Bayesian criterion
CCF: Cross Correlation function
