# Supplementary figures and images for: Effects of meteorological factors on the incidence of mumps and models for prediction, China
Source: BMC Infect Dis. 2020 Jul 2;20:468. doi: 10.1186/s12879-020-05180-7 (PMC7331163; doi:10.1186/s12879-020-05180-7)

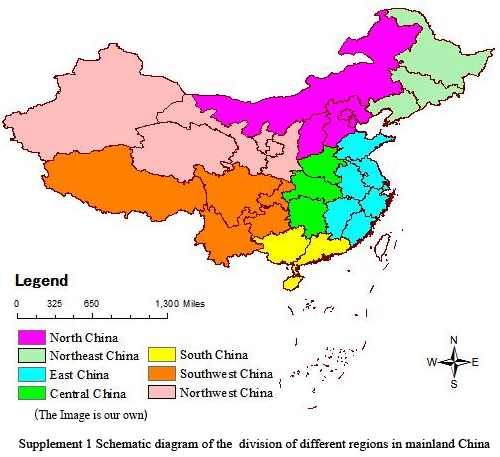

Supplement: Supplementary file 1 — Additional file 1: Supplement 1. Schematic diagram of the division of different regiousnin mainland China. [file 12879_2020_5180_MOESM1_ESM.tif]

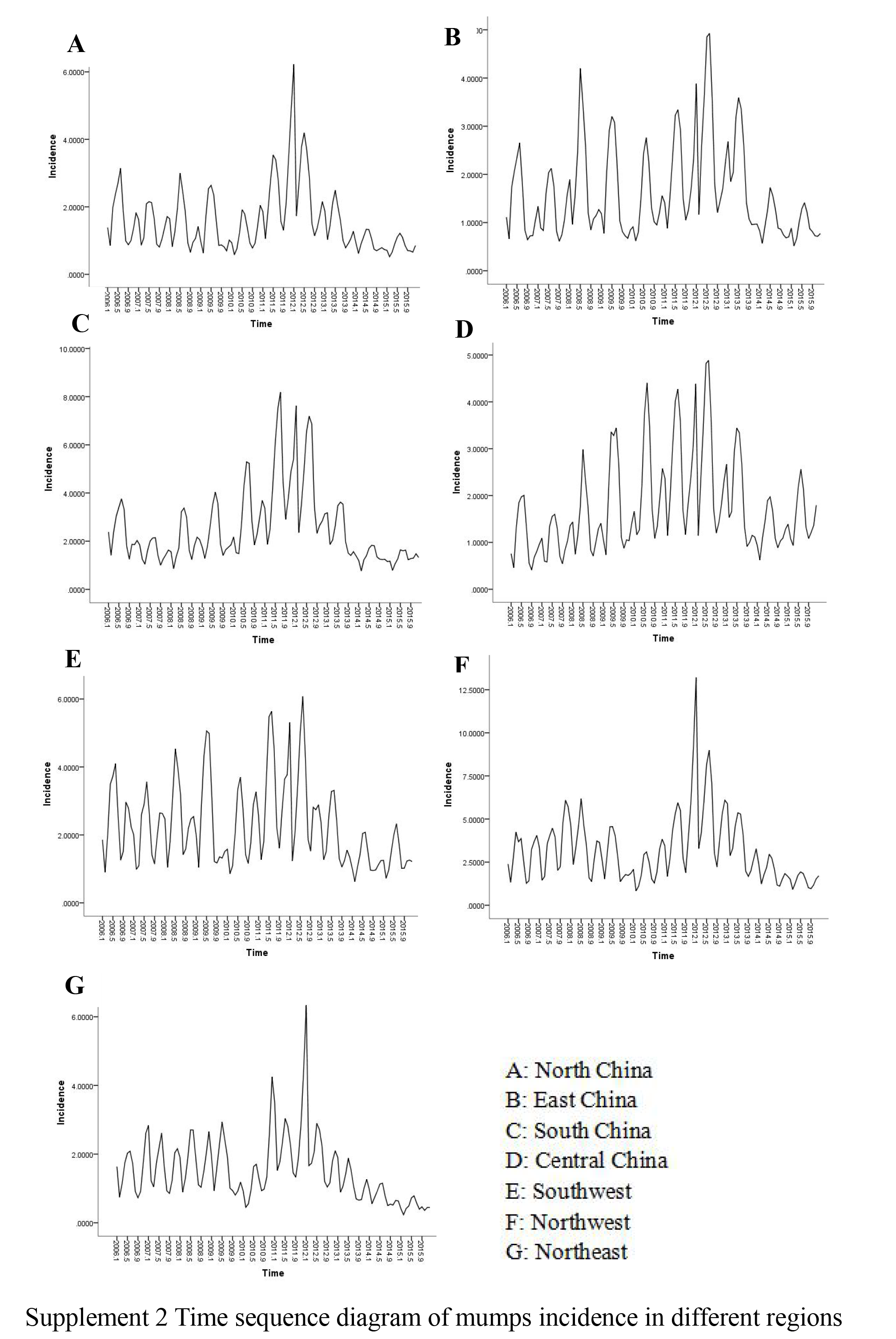

Supplement: Supplementary file 2 — Additional file 2: Supplement 2. Time sequence diagram of mumps incidence in different regions. [file 12879_2020_5180_MOESM2_ESM.tif]
