# Supplementary material for: Effects of meteorological factors on the incidence of mumps and models for prediction, China
Source: BMC Infect Dis. 2020 Jul 2;20:468. doi: 10.1186/s12879-020-05180-7 (PMC7331163; doi:10.1186/s12879-020-05180-7)
Supplement: Supplementary file 3 — Additional file 3: Supplement 3. The optimal ARIMAX model for each meteorological sequence in different regions of China. [file 12879_2020_5180_MOESM3_ESM.doc]

Supplement 3 The optimal ARIMAX model for each meteorological sequence in different regions of China

| Regions | Meteorological factors | Model | Fitting effect | | |  | LBQ test | | |
| --- | --- | --- | --- | --- | --- | --- | --- | --- | --- |
|  |  |  | R^2^ | Stable R^2^ | SBC |  | Q | P | |
| North China | A | (0,0,0)(0,1,1)_12_ | 0.683 | 0.293 | 6.617 |  | 8.354 | | 0.958 |
|  | B | (0,0,0)(0,1,1)_12_ | 0.914 | 0.347 | 1.513 |  | 18.101 | | 0.382 |
|  | C | (0,0,1)(2,1,0)_12_ | 0.985 | 0.405 | 0.779 |  | 15.161 | | 0.440 |
|  | D | (1,0,0)(1,1,0)_12_ | 0.532 | 0.255 | 3.929 |  | 22.078 | | 0.141 |
|  | E | (0,0,0)(1,1,0)_12_ | 0.983 | 0.247 | 0.750 |  | 20.300 | | 0.259 |
|  | F | (3,1,0)(2,1,0)_12_ | 0.976 | 0.595 | 1.332 |  | 12.710 | | 0.470 |
|  | G | (0,0,0)(0,1,1)_12_ | 0.678 | 0.295 | 1.590 |  | 14.267 | | 0.648 |
|  | H | (1,0,0)(0,1,1)_12_ | 0.254 | 0.346 | 0305 |  | 18.810 | | 0.279 |
| East China | A | (0,0,0)(1,1,1)_12_ | 0.592 | 0.452 | 7.615 |  | 12.303 | | 0.723 |
|  | B | (0,0,0)(2,1,0)_12_ | 0.939 | 0.283 | 1.419 |  | 23.727 | | 0.096 |
|  | C | (0,0,0)(2,1,0)_12_ | 0.973 | 0.311 | 0.757 |  | 5.512 | | 0.993 |
|  | D | (1,1,1)(1,0,1)_12_ | 0.291 | 0.450 | 3.384 |  | 19.139 | | 0.160 |
|  | E | (0,0,0)(1,1,0)_12_ | 0.973 | 0.243 | 0.685 |  | 13.911 | | 0.673 |
|  | F | (0,1,1)(2,1,0)_12_ | 0.956 | 0.602 | 1.306 |  | 7.185 | | 0.952 |
|  | G | (0,0,0)(0,1,2)_12_ | 0.279 | 0.519 | 2.132 |  | 20.114 | | 0.215 |
|  | H | (0,1,1)(1,0,1)_12_ | 0.258 | 0.430 | 0.022 |  | 23.083 | | 0.082 |
| South China | A | (0,0,0)(0,1,1)_12_ | 0.517 | 0.303 | 8.933 |  | 26.670 | | 0.063 |
|  | B | (1,0,1)(1,1,0)_12_ | 0.910 | 0.273 | 1.330 |  | 22.044 | | 0.107 |
|  | C | (0,0,0)(3,1,0)_12_ | 0.941 | 0.415 | 0.778 |  | 12.637 | | 0.630 |
|  | D | (0,0,0)(0,1,1)_12_ | 0.378 | 0.406 | 2.937 |  | 17.403 | | 0.427 |
|  | E | (0,0,0)(2,1,0)_12_ | 0.941 | 0.415 | 0.662 |  | 21.983 | | 0.144 |
|  | F | (0,1,1)(2,1,0)_12_ | 0.904 | 0.650 | 1.349 |  | 12.639 | | 0.630 |
|  | G | (0,0,0)(0,1,1)_12_ | 0.434 | 0.399 | 2.537 |  | 20.133 | | 0.268 |
|  | H | (0,1,1)(1,0,1)_12_ | 0.356 | 0.264 | 0.571 |  | 12.572 | | 0.635 |
| Central China | A | (0,0,0)(1,1,1)_12_ | 0.554 | 0.543 | 7.368 |  | 7.631 | | 0.959 |
|  | B | (0,1,1)(1,1,0)_12_ | 0.899 | 0.553 | 2.096 |  | 18.700 | | 0.285 |
|  | C | (0,0,0)(2,1,0)_12_ | 0.972 | 0.287 | 0.885 |  | 7.554 | | 0.961 |
|  | D | (1,1,1)(1,0,1)_12_ | 0.226 | 0.351 | 3.649 |  | 18.349 | | 0.191 |
|  | E | (0,0,0)(0,1,1)_12_ | 0.976 | 0.289 | 0.662 |  | 13.812 | | 0.680 |
|  | F | (0,0,0)(0,1,1)_12_ | 0.958 | 0.337 | 1.242 |  | 7.839 | | 0.970 |
|  | G | (0,0,0)(3,1,0)_12_ | 0.069 | 0.406 | 2.272 |  | 24.481 | | 0.057 |
|  | H | (0,1,1)(0,0,0)_12_ | 0.507 | 0.241 | 0.457 |  | 8.356 | | 0.958 |
| Southwest | A | (1,0,1)(0,1,1)_12_ | 0.705 | 0.315 | 7.217 |  | 11.059 | | 0.748 |
|  | B | (0,0,0)(2,1,0)_12_ | 0.601 | 0.275 | 1.871 |  | 7.050 | | 0.972 |
|  | C | (0,0,0)(2,1,0)_12_ | 0.972 | 0.364 | -0.010 |  | 9.512 | | 0.891 |
|  | D | (1,0,0)(0,1,1)_12_ | 0.831 | 0.375 | 2.491 |  | 7.620 | | 0.959 |
|  | E | (0,0,0)(0,1,1)_12_ | 0.984 | 0.371 | -0.518 |  | 10.530 | | 0.880 |
|  | F | (0,0,0)(2,1,0)_12_ | 0.952 | 0.367 | 0.486 |  | 13.341 | | 0.658 |
|  | G | (0,0,0)(0,1,1)_12_ | 0.656 | 0.247 | 1.929 |  | 19.685 | | 0.291 |
|  | H | (1,0,1)(0,1,1)_12_ | 0.561 | 0.341 | -0.361 |  | 12.517 | | 0.640 |
| Northwest | A | (0,0,0)(0,1,1)_12_ | 0.728 | 0.235 | 5.820 |  | 21.831 | | 0.191 |
|  | B | (0,0,0)(2,1,0)_12_ | 0.895 | 0.272 | 1.076 |  | 19.302 | | 0.253 |
|  | C | (0,0,0)(3,1,0)_12_ | 0.981 | 0.365 | 0.879 |  | 11.030 | | 0.750 |
|  | D | (0,0,0)(1,1,0)_12_ | 0.513 | 0.322 | 3.500 |  | 21.939 | | 0.187 |
|  | E | (0,0,0)(3,1,0)_12_ | 0.982 | 0.373 | 0.753 |  | 8.633 | | 0.896 |
|  | F | (0,0,0)(3,1,0)_12_ | 0.970 | 0.372 | 1.318 |  | 18.536 | | 0.236 |
|  | G | (0,0,0)(2,1,0)_12_ | 0.589 | 0.360 | 1.633 |  | 23.210 | | 0.108 |
|  | H | (0,0,1)(1,0,1)_12_ | 0.294 | 0.294 | 0.307 |  | 17.896 | | 0.268 |
| Northeast | A | (0,0,0)(2,1,0)_12_ | 0.630 | 0.392 | 6.968 |  | 10.494 | | 0.840 |
|  | B | (0,0,0)(1,1,0)_12_ | 0.871 | 0.237 | 2.004 |  | 14.147 | | 0.657 |
|  | C | (0,0,1)(3,1,0)_12_ | 0.980 | 0.400 | 1.552 |  | 22.106 | | 0.076 |
|  | D | (0,1,1)(2,1,1)_12_ | 0.632 | 0.673 | 3.674 |  | 13.015 | | 0.525 |
|  | E | (1,0,0)(2,1,0)_12_ | 0.983 | 0.396 | 1.314 |  | 20.830 | | 0.142 |
|  | F | (1,1,1)(2,1,0)_12_ | 0.974 | 0.582 | 1.803 |  | 19.893 | | 0.134 |
|  | G | (0,0,0)(2,1,0)_12_ | 0.347 | 0.343 | 2.382 |  | 9.687 | | 0.882 |
|  | H | (1,0,0)(0,0,0)_12_ | 0.016 | 0.052 | 1.667 |  | 25.061 | | 0.093 |

Note：A: Average precipitation, B: Average air pressure, C: Average temperature, D: Average relative humidity, E: Minimum temperature, F: Maximum temperature, G: Days with daily drecipitation ≥0.1mm, H: Maximum wind speed
